# The value of FDG combined with PiB PET in the diagnosis of patients with cognitive impairment in a memory clinic

**DOI:** 10.1111/cns.14418

**Published:** 2023-08-21

**Authors:** Fang Liu, Yudi Shi, Qiuyan Wu, Huifeng Chen, Ying Wang, Li Cai, Nan Zhang

**Affiliations:** ^1^ Department of Neurology Tianjin Neurological Institute, Tianjin Medical University General Hospital Tianjin China; ^2^ Health Management Center Tianjin Medical University General Hospital Airport Site Tianjin China; ^3^ Department of Neurology Tianjin Medical University General Hospital Airport Site Tianjin China; ^4^ PET/CT Center Tianjin Medical University General Hospital Tianjin China

**Keywords:** cognitive impairment, fluorodeoxyglucose, memory clinic, Pittsburgh compound B, positron emission tomography

## Abstract

**Aims:**

To analyze the value of ^18^F‐fluorodeoxyglucose (FDG) positron emission tomography (PET) combined with amyloid PET in cognitive impairment diagnosis.

**Methods:**

A total of 187 patients with dementia or mild cognitive impairment (MCI) who underwent ^11^C‐Pittsburgh compound B (PiB) and FDG PET scans in a memory clinic were included in the final analysis.

**Results:**

Amyloid‐positive and amyloid‐negative dementia patient groups showed a significant difference in the proportion of individuals presenting temporoparietal cortex (*p* < 0.001) and posterior cingulate/precuneus cortex (*p* < 0.001) hypometabolism. The sensitivity and specificity of this hypometabolic pattern for identifying amyloid pathology were 72.61% and 77.97%, respectively, in patients clinically diagnosed with AD and 60.87% and 76.19%, respectively, in patients with MCI. The initial diagnosis was changed in 32.17% of patients with dementia after considering both PiB and FDG results. There was a significant difference in both the proportion of patients showing the hypometabolic pattern and PiB positivity between dementia conversion patients and patients with a stable diagnosis of MCI (*p* < 0.05).

**Conclusion:**

Temporoparietal and posterior cingulate/precuneus cortex hypometabolism on FDG PET suggested amyloid pathology in patients with cognitive impairment and is helpful in diagnostic decision‐making and predicting AD dementia conversion from MCI, particularly when combined with amyloid PET.

## Graphical abstract

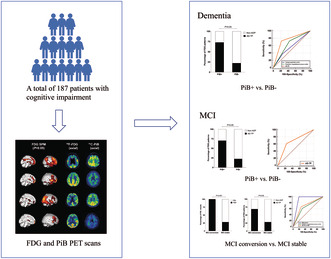

## 1 INTRODUCTION

According to the reports of the Global Burden of Disease Study 2019, the number of people with dementia was projected to increase from 57.4 million globally in 2019 to 152.8 million in 2050.^1^ However, diagnosis of dementia can be challenging, particularly in patients with early stages of dementia, young age of onset, atypical/unclear presentations, or comorbid neuropsychiatric symptomatology.^2^, ^3^ As disease‐modifying therapies (DMTs) targeting the underlying pathophysiology are emerging, accurate and differential diagnosis have become increasingly important for facilitating etiology‐specific treatment in patients with dementia. Positron emission tomography (PET) neuroimaging techniques play an important role in clinical diagnosis and have been recommended for use in the clinical diagnosis of neurodegenerative dementia with appropriate tracers to assess pathology and pathophysiology in vivo.^4^, ^5^, ^6^, ^7^ Compared with indirect measurement of abnormal molecular expression in the cerebral spinal fluid (CSF), PET scans are noninvasive and able to reflect the topographical distribution of misfolded protein deposition and metabolic changes. Although novel tracers for tau and neuroinflammation have been approved and investigated, respectively, for dementia diagnosis, amyloid and fluorine‐18‐fluorodeoxyglucose (^18^F‐FDG) tracers are still the most commonly used and available in clinical practice.

Amyloid PET ligands, such as ^18^F‐florbetapir, ^18^F‐florbetaben, and ^18^F‐flutemetamol, which detect and quantify amyloid neurotic plaques in vivo, have been approved for the diagnosis of Alzheimer's disease (AD) by the US FDA. Previously, carbon‐11‐labeled Pittsburgh compound B (^11^C‐PiB), the first tracer specific to β‐amyloid (Aβ) applied in human studies,^8^ was found to bind with high affinity to fibrillar Aβ deposits in pathological studies.^9^, ^10^

Cerebral hypometabolism measured with FDG PET, which reflects synaptic failure at the cellular level, is a downstream marker indicating neuronal injury and neurodegeneration^11^, ^12^ and has been included in the AD research framework^4^ and in diagnostic criteria for other types of dementia, according to the characteristic hypometabolic locations or patterns, such as predominant posterior cingulate and temporoparietal cortex in typical AD, frontal and anterior temporal hypometabolism in frontotemporal lobar degeneration (FTLD),^5^, ^6^ and occipital hypometabolism with or without the cingulate island sign in dementia with Lewy bodies (DLB).^7^ Based on a literature search using the population, intervention, comparison, and outcome (PICO) model, a previous study used the Delphi method to determine that FDG PET was good for discriminating DLB from AD (sensitivity: 70%–92%, specificity: 74%–100%), fair for discriminating FTLD from AD (sensitivity: 80%–99%, specificity: 63%–98%), and lacking for discriminating other types of dementia.^13^

Compared with FDG, PiB had higher sensitivity (96% vs. 80%) and similar specificity (86% vs. 84%) for detecting AD neuropathologic changes in autopsy‐confirmed participants, and the sensitivity and specificity approached 97% and 98%, respectively, when both imaging modalities were congruent.^14^ Moreover, in a cohort with cognitive impairment, it was reported that in 23% of cases, neurologists changed their initial diagnosis after considering FDG and PiB PET results, with the combination of PiB and FDG contributing to the most changed diagnoses (68%), followed by PiB only (28%) and FDG only (7%).^15^

Therefore, although amyloid PET has a good performance in predicting AD pathology, FDG PET shows additional value in increasing the diagnostic accuracy, differentiating with other forms of dementia, and influencing subsequent diagnosis and treatment decisions. In this study, the positivity of PiB PET and the hypometabolic pattern of FDG PET were retrospectively analyzed in patients with dementia or mild cognitive impairment (MCI) being treated at a memory clinic. The diagnostic accuracies of FDG PET for both AD and MCI due to AD were identified based on the amyloid positivity of PiB PET. The value of combining PiB and FDG PET scans on the diagnostic process was further summarized.

## 2 MATERIALS AND METHODS

### 2.1 Participants

Detailed written informed consent was obtained from all subjects and their relatives. A total of 201 consecutive patients with cognitive impairment who visited the memory clinic and received PiB and FDG PET scans at Tianjin Medical University General Hospital between July 2014 and August 2021 were retrospectively reviewed. All patients also underwent a comprehensive evaluation, such as a medical history collection based on both subject and caregiver interviews, physical and neurological examinations, laboratory tests (e.g., thyroid function, vitamin B12, folate, and syphilis serology), brain magnetic resonance imaging (MRI), and neuropsychological assessments. After careful review of the medical records of all participants, 187 patients were included in the final analysis and 12 patients without detailed clinical data were excluded.

All participants were diagnosed by dementia specialists at the memory clinic according to specific diagnostic criteria, such as the revised National Institute on Aging and the Alzheimer's Association (NIA‐AA) criteria for probable AD dementia,^16^ Petersen's criteria for MCI,^17^ the revised Frontotemporal Dementia Consensus criteria for behavioral variant frontotemporal dementia (bvFTD),^5^ the classification recommendations for primary progressive aphasia (PPA),^6^ the Third or Fourth report of DLB Consortium for probable DLB,^7^, ^18^ the clinical criteria for corticobasal syndrome (CBS),^19^ the Vascular Behavioral and Cognitive Disorders criteria for vascular dementia (VaD),^20^ and the fifth edition of the diagnostic and statistical manual of mental disorders (DSM‐5) criteria for depression.^21^

For further analysis, persons with dementia and persons with MCI were divided into two groups, with persons with depression included in the dementia group because they presented pseudodementia.

### 2.2 PET imaging

PiB and FDG PET scans were conducted at the PET/CT center of Tianjin Medical University General Hospital on a Discovery PET/CT 710 scanner (GE Healthcare) in the three‐dimensional scanning mode. PiB was injected into an antecubital vein as a bolus injection, with a mean dose of 370–555 MBq. Images were acquired during a 90‐min dynamic PET scan (34 frames: 4 × 15 s, 8 × 30 s, 9 × 60 s, 2 × 180 s, 8 × 300 s, 3 × 600 s). At a minimum of 1 h after PiB injection, subjects were intravenously injected with 259 MBq of FDG and then received a 10‐min static PET scan at 40 mins after FDG injection. Each frame produced 47 slices with 3.75 mm thickness, which covered the whole brain. Both PiB and FDG images were reconstructed to a 256 × 256 matrix (pixel size of 1.37 mm^2^).

### 2.3 PET interpretation

Positron emission tomography scans were visually read by two experienced nuclear medicine physicians (Li Cai and Ying Wang) according to a procedure described in our previous publications.^22^, ^23^ The mean values for all the specific regions were calculated from the integral PiB image. The positivity or negativity of PiB PET was determined by the ratio of the mean value of the target region to that of the cerebellum with a cutoff value of 1.5 (the upper 95% confidence interval from a cluster analysis of healthy individuals).

Fluorodeoxyglucose frames for each subject were summed and normalized to the mean activity in the pons, then were presented in the NIH color scale and could be windowed and viewed in three planes at the rater's discretion. In addition to examination of specific brain regions, such as the medial frontal lobes, lateral frontal lobes, anterior temporal cortex, temporoparietal cortex, posterior cingulate/precuneus cortices, and occipital lobe, the results of FDG PET images were further classified into two patterns according to their hypometabolic topography: the “AD‐typical pattern” was defined as hypometabolism that was mostly observed in the temporoparietal cortex and posterior cingulate cortex and the “non‐AD pattern” was characterized by either hypometabolism mainly in other brain areas or nonsignificant hypometabolism relative to normal controls.

### 2.4 Follow‐up

Diagnostic changes after considering PET results were recorded for all patients with dementia. For patients with MCI, long‐term follow‐up was performed, mainly including medical histories and neuropsychological testing, to determine the conversion to dementia, which was diagnosed according to the criteria for major neurocognitive disorder of DSM‐5^21^ and diagnostic criteria for specific diseases or syndromes mentioned above.

### 2.5 Statistical analysis

Continuous variables were expressed as the mean ± standard deviation. Categorical data were expressed as numbers and percentages. The differences in proportions of hypometabolic regions and hypometabolic patterns between amyloid‐positive persons and amyloid‐negative persons were examined with chi‐squared tests for the dementia group and MCI group, respectively. Receiver operating characteristic (ROC) curves were used to determine the performance of FDG PET in predicting AD pathology in patients with dementia or MCI according to the PiB PET results. Chi‐squared tests were used to examine the differences in proportions of hypometabolic regions and hypometabolic patterns between persons with MCI remaining stable and those converting to dementia. *p* values <0.05 were considered statistically significant.

## 3 RESULTS

### 3.1 Demographic and clinical characteristics and results of PiB PET

Participants were initially diagnosed with AD (*n* = 94, including 92 participants with typical AD and two with posterior variant of AD), MCI (*n* = 44), FTLD (*n* = 25, including 14 participants with bvFTD, six with a semantic variant of PPA [svPPA], four with a nonfluent variant of PPA [nfvPPA], and one with CBS), DLB (*n* = 3), VaD (*n* = 3, including one patient with VaD and two with mixed dementia), depression (*n* = 6), and dementia of unclear etiology (not otherwise specified [Dem NOS], *n* = 12) before PET scan measurements. There were 107 patients presenting a positive result on PiB PET (Table 1). The proportions of amyloid positivity were 72.34% (68/94), 52.27% (23/44), 28.00% (7/25), 66.67% (2/3), 66.67% (2/3), 16.67% (1/6), and 50% (6/12) in AD, MCI, FTLD, DLB, VaD, depression, and Dem NOS patients, respectively.

**TABLE 1:** Demographic and clinical characteristics according to the initial clinical diagnosis prior to PET assessment.

	AD (*n* = 94)^a^	MCI (*n* = 44)	FTLD (*n* = 25)			DLB (*n* = 3)	VaD (*n* = 3)^c^	Depression (*n* = 6)	Dem NOS (*n* = 12)
			bvFTD (*n* = 14)	PPA (*n* = 10)^b^	CBS (*n* = 1)				
Age, years	67.1 ± 8.7	67.8 ± 6.9	63.7 ± 7.3	64.2 ± 7.0	66	65.3 ± 7.6	72.0 ± 4.0	61.5 ± 10.8	68.3 ± 7.4
Sex, M/F	34/60	12/32	5/9	4/6	1/0	2/1	2/1	1/5	5/7
Education, years	10.8 ± 3.9	11.1 ± 3.9	10.6 ± 1.9	11.4 ± 2.9	9	10.0 ± 1.7	13.3 ± 2.3	9.5 ± 5.8	9.1 ± 5.2
Amyloid positivity, *n* (%)	68 (72.34)	23 (52.27)	5 (35.71)	2 (20.00)	0	2 (66.67)	2 (66.67)	1 (16.67)	6 (50.00)

Data are presented as the mean ± SD for age and education except CBS group (*n* = 1).

Abbreviations: AD, Alzheimer's disease; bvFTD, behavioral variant frontotemporal dementia; CBS, corticobasal syndrome; Dem NOS, dementia not otherwise specified; DLB, dementia with Lewy bodies; FTLD, frontotemporal lobar degeneration; MCI, mild cognitive impairment; PPA, primary progressive aphasia; VaD, vascular dementia.

^a^ Included 92 patients with typical AD and two patients with posterior variant of AD.

^b^ Included six patients with semantic variant of PPA and four patients with nonfluent variant of PPA.

^c^ Included one patient with VaD and two patients with mixed dementia (VaD plus AD).

### 3.2 The performance of hypometabolic regions and patterns on FDG in predicting amyloid deposition in patients with dementia or MCI

**FIGURE 1:**
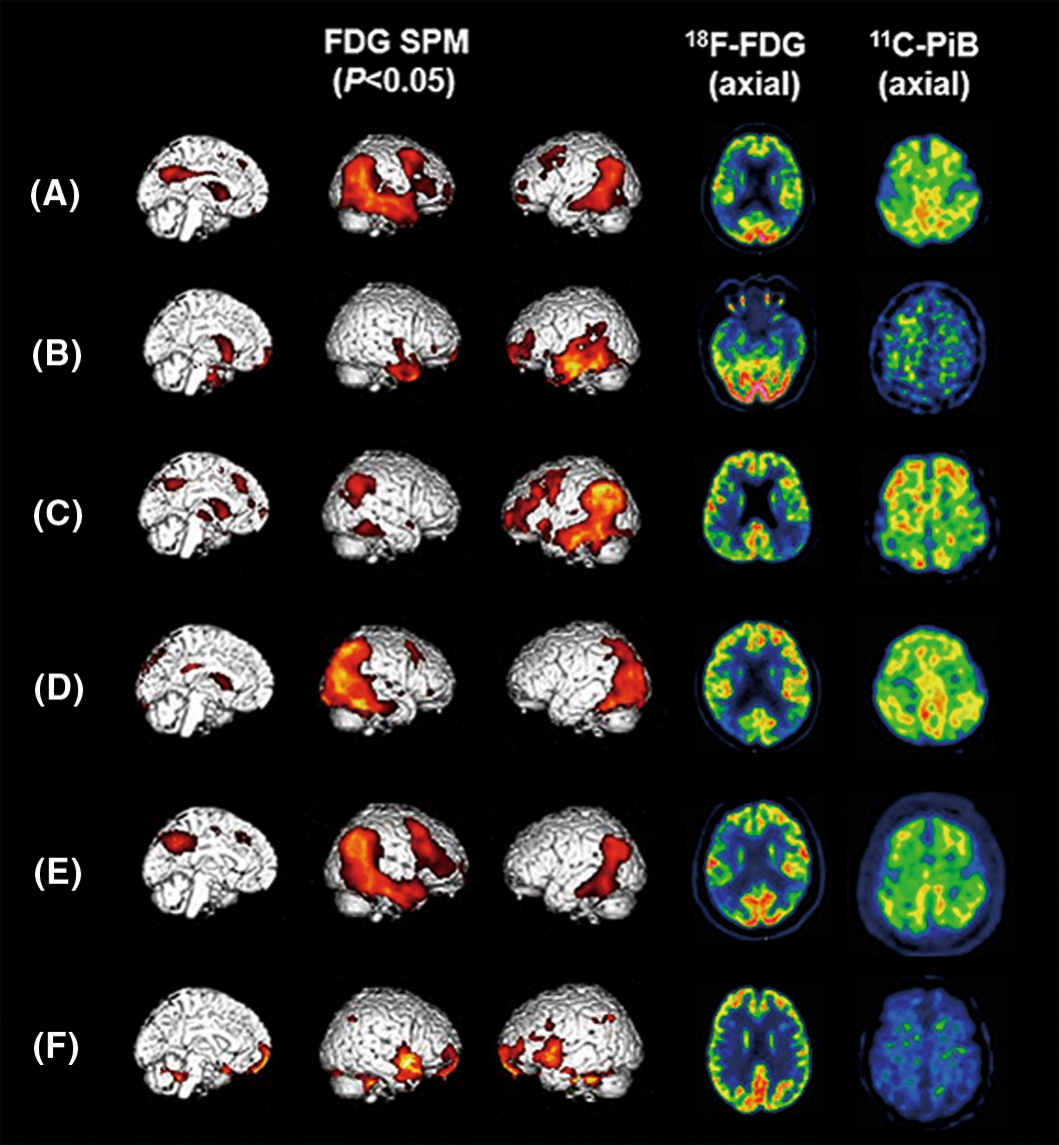
FDG‐PET and PiB‐PET images of six representative participants. (A) An AD patient with an “AD‐typical pattern” on the FDG‐PET image featuring predominant hypometabolism in the temporoparietal cortex and posterior cingulate cortex and with a positive PiB‐PET result. (B) A patient with a semantic variant of PPA due to FTLD who was initially diagnosed with AD before PET examination showed hypometabolism in the anterior temporal lobe and prefrontal lobes (“non‐AD pattern”) on the FDG image and had negative PiB‐PET results. (C) A patient with logopenic variant of AD who was initially diagnosed with a nonfluent variant of PPA before PET examination showed an “AD‐typical pattern” but left predominant hypometabolism on the FDG image and had positive PiB results. (D) A patient with posterior variant of AD who was initially diagnosed with DLB before PET examination showed hypometabolism in the occipital lobes and posterior parietal lobes on the FDG image and had positive PiB results. (E) An MCI patient converted to AD dementia 14 months after PET examination and showed an “AD‐typical pattern” on the FDG image with positive PiB results. (F) An MCI patient remained stable 17 months after PET examination and showed a “non‐AD pattern” with nonspecific hypometabolism in a few temporal and frontal areas on the FDG image and had negative PiB results. AD, Alzheimer's disease; DLB, dementia with Lewy bodies; FDG, fluorodeoxyglucose; MCI, mild cognitive impairment; PET, positron emission tomography; PiB, Pittsburgh compound B; PPA, primary progressive.

**FIGURE 2:**
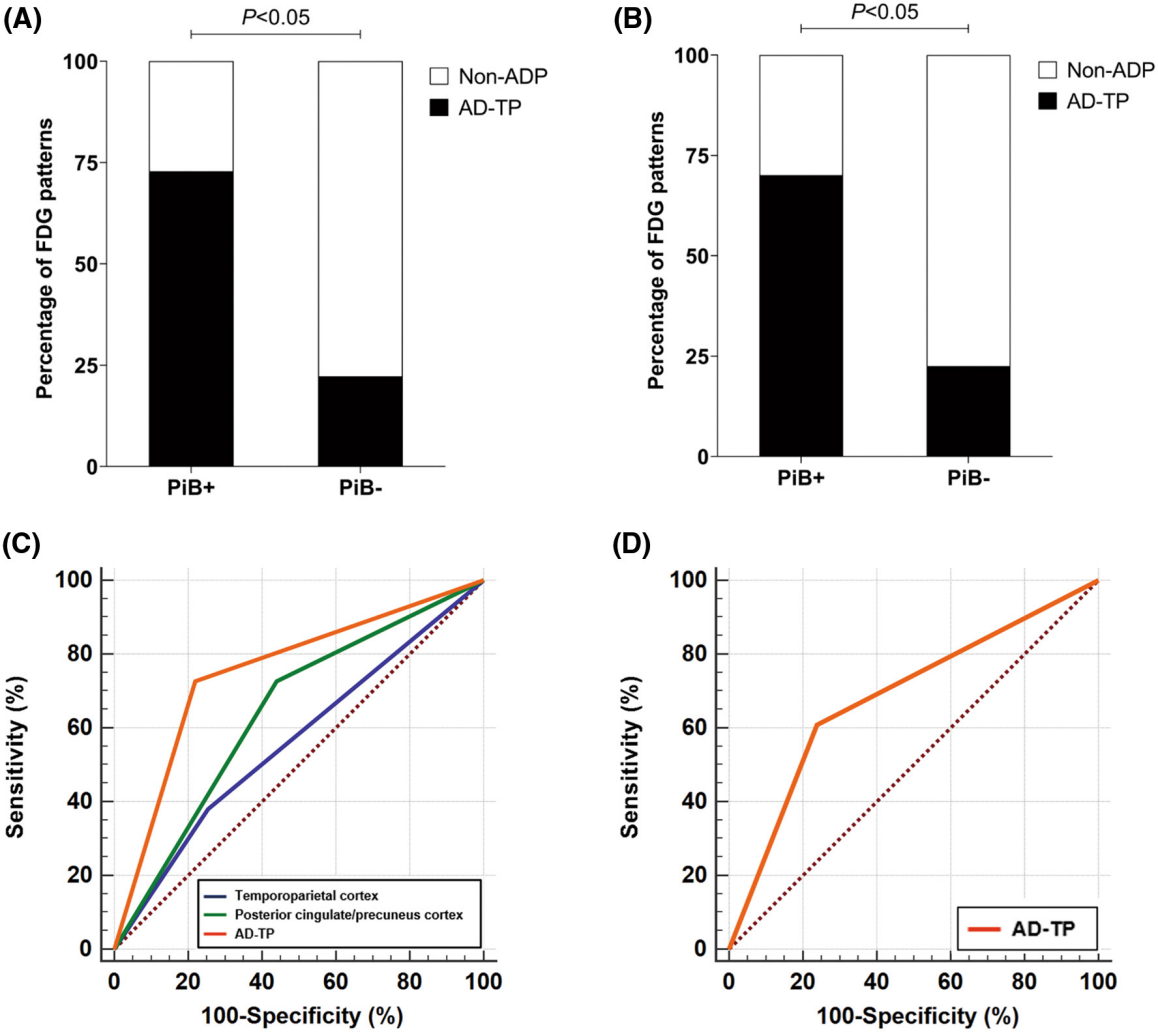
Hypometabolic regions and patterns of FDG in patients with cognitive impairment. There was a significant difference in the hypometabolic pattern (%) between amyloid‐positive (PiB+) and amyloid‐negative (PiB‐) patients with dementia (A) or patients with MCI (B). According to ROC curves, hypometabolism in the posterior cingulate/precuneus cortex (sensitivity: 72.62%, specificity: 55.93%, PPV: 79.31%, NPV: 73.21%, AUC: 0.643, *p* < 0.001) and an “AD‐typical pattern” (sensitivity: 72.61%, specificity: 77.97%, PPV: 86.59%, NPV: 71.95%, AUC: 0.753, *p* < 0.001), but not hypometabolism in the temporoparietal cortex (sensitivity: 38.10%, specificity: 74.58%, PPV: 89.36%, NPV: 56.25%, AUC: 0.563, *p* = 0.105), effectively discriminated dementia patients with amyloid‐positive and amyloid‐negative features (C); and hypometabolism in an “AD‐typical pattern” (sensitivity: 60.87%, specificity: 76.19%, PPV: 73.68%, NPV: 64.00%, AUC: 0.685, *p* < 0.05) could discriminate MCI patients with amyloid‐positive and amyloid‐negative features (D). AD, Alzheimer's disease; AD‐TP, AD‐typical pattern; AUC, area under the curve; FDG, fluorodeoxyglucose; MCI, mild cognitive impairment; Non‐ADP, non‐AD pattern; NPV: negative predictive value; PiB, Pittsburgh compound B; PPV: positive predictive value; ROC, receiver operating characteristic.

Figure 1 shows representative FDG and PiB images of four participants with dementia and two participants with MCI. Compared with amyloid‐negative patients, a higher proportion of amyloid‐positive patients had hypometabolism in the temporoparietal cortex (*p* < 0.001) and posterior cingulate/precuneus cortex (*p* < 0.001) in the dementia group; no statistically significant difference in the hypometabolic region was observed in the MCI group (Table S1). There was a significant difference in the proportion of hypometabolic patterns between amyloid‐positive and amyloid‐negative patients with dementia (*p* < 0.05) and MCI (*p* < 0.05). Specifically, the “AD‐typical pattern” was shown in 61 (72.62%) dementia patients and 14 (60.87%) MCI patients with a positive result on PiB PET and in 13 (22.03%) dementia patients and five (23.81%) MCI patients with a negative result on PiB PET (Figure 2A,B).

Receiver operating characteristic curve analysis was performed for hypometabolic regions and patterns on FDG with significant differences in group comparison, taking PiB positivity as the gold standard for AD diagnosis (Figure 2C,D). Compared with regional hypometabolism in the temporoparietal cortex (area under the ROC curve [AUC]: 0.563, sensitivity: 38.10%, specificity: 74.58%) and posterior cingulate/precuneus cortex (AUC: 0.643, sensitivity: 72.62%, specificity: 55.93%), the “AD‐typical pattern” on FDG PET had a higher AUC of 0.753 (*p* < 0.05), with sensitivity and specificity of 72.61% and 77.97% for identifying AD pathology in dementia patients. In terms of MCI patients, the “AD‐typical pattern” on FDG PET showed an AUC of 0.685 (*p* < 0.05), with a sensitivity and specificity of 60.87% and 76.19%, respectively.

### 3.3 Diagnostic changes after PET assessment in patients with dementia

The initial diagnosis was changed after considering PET results in 32.17% of patients with dementia. Clinically diagnosed patients with AD and other types of dementia were stratified into four groups based on both PiB and FDG PET results: PiB+ and AD‐typical pattern, PiB+ and non‐AD pattern, PiB‐ and AD‐typical pattern, and PiB‐ and non‐AD pattern. Table 2 shows the diagnostic changes before and after PET assessments in all groups. For clinically diagnosed AD patients, all 49 patients with PiB+ and AD‐typical patterns maintained their original AD diagnosis; the diagnoses of two of 19 patients with PiB+ and non‐AD patterns were changed to DLB or svPPA (each *n* = 1); the diagnoses of 10 of 11 patients with PiB‐ and AD‐typical patterns were changed to bvFTD (*n* = 3), svPPA (*n* = 1), VaD (*n* = 2), encephalopathia alcoholica (*n* = 1), or Dem NOS (*n* = 3), respectively; the diagnoses of 14 of 15 patients with PiB‐ and non‐AD patterns were changed to bvFTD (*n* = 5), svPPA (*n* = 1), depression (*n* = 3), and Dem NOS (*n* = 5), respectively.

**TABLE 2:** Diagnostic changes in dementia patients stratified by FDG and PiB PET results.

Initial clinical diagnosis	PET results	Patients with changed diagnosis at follow‐up/total number	Specific changes in diagnosis
AD	PiB+/AD pattern	0/49	NA
	PiB+/non‐AD pattern	2/19	1 DLB, 1 svPPA
	PiB‐/AD pattern	10/11	3 bvFTD, 1 svPPA, 2 VaD, 1 encephalopathia alcoholica, 3 Dem NOS
	PiB‐/non‐AD pattern	14/15	5 bvFTD, 1 svPPA, 3 depression, 5 Dem NOS
Non‐AD	PiB+/AD pattern	12/12	1 DLB, 4 bvFTD, 1 nfvPPA and 6 Dem NOS to AD^a^
	PiB+/non‐AD pattern	4/4	1 bvFTD, 1 DLB and 1 Dem NOS to AD^b^, 1 depression to Dem NOS
	PiB‐/AD pattern	1/2	1 Dem NOS to VaD
	PiB/non‐AD pattern	3/31	1 VaD to DLB^c^, 1 Dem NOS to bvFTD

Abbreviations: AD, Alzheimer's disease; bvFTD, behavioral variant frontotemporal dementia; Dem NOS, dementia not otherwise specified; DLB, dementia with Lewy bodies; FDG, fluorodeoxyglucose; NA, not applicable; nfvPPA, non‐fluent variant of primary progressive aphasia; PET, positron emission tomography; PiB, Pittsburgh compound B; svPPA, semantic variant of primary progressive aphasia; VaD, vascular dementia.

^a^ One bvFTD patient was revised to a diagnosis of frontal variant of AD and the nfvPPA patient was revised to a diagnosis of logopenic variant of AD.

^b^ The bvFTD patient was revised to a diagnosis of frontal variant of AD and the DLB patient was revised to diagnosis of posterior variant of AD.

^c^ Two patients with mixed VaD were excluded to comorbid with AD pathology.

Considering patients with an initial diagnosis of non‐AD dementia, the diagnoses of all 12 patients with PiB+ and AD‐typical patterns were revised to AD, with one bvFTD and one nfvPPA patient finally being diagnosed as frontal variant of AD (fvAD) and logopenic variant of PPA (lvPPA), respectively; in four patients with PiB+ and non‐AD patterns, the diagnosis of one bvFTD patient was changed to fvAD, one DLB patient to posterior variant of AD, one Dem NOS patient to AD, and one patient with depression to Dem NOS; in two patients with PiB‐ and AD‐typical patterns, one patient maintained their original bvFTD diagnosis and one Dem NOS patient's diagnosis was changed VaD; in 31 patients with PiB‐ and non‐AD patterns, the diagnosis of one patient with VaD was changed to DLB, the diagnoses of two patients with mixed VaD were excluded to comorbid with AD pathology, one Dem NOS patient's diagnoses was changed to bvFTD, and the other patients maintained their original diagnosis.

### 3.4 Prediction of conversion to dementia from MCI for PiB and FDG PET

**FIGURE 3:**
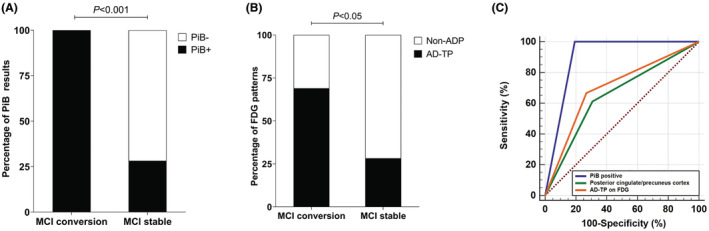
PiB and FDG results in MCI patients with different clinical outcomes. There was a significant difference in (A) PiB positivity (%) and (B) hypometabolic pattern on FDG images (%) between MCI patients converting to AD dementia and MCI patients remaining stable. According to ROC curves, positive results on PiB (sensitivity: 100.00%, specificity: 80.77%, PPV: 69.57%, NPV: 100.00%, AUC: 0.904, *p* < 0.001), hypometabolism in the posterior cingulate/precuneus cortex (sensitivity: 61.11%, specificity: 69.23%, PPV: 64.71%, NPV: 79.17%, AUC: 0.652, *p* < 0.05) and an “AD‐typical pattern” (sensitivity: 66.67%, specificity: 73.08%, PPV: 61.11%, NPV: 78.26%, AUC: 0.699, *p* < 0.05), efficiently discriminated MCI patients converting to AD dementia from those remaining stable (C). AD, Alzheimer's disease; AD‐TP, AD‐typical pattern; AUC, area under the curve; FDG, fluorodeoxyglucose; MCI, mild cognitive impairment; Non‐ADP, non‐AD pattern; NPV: negative predictive value; PiB, Pittsburgh compound B; PPV: positive predictive value; ROC, receiver operating characteristic.

At long‐term follow‐up (median time 32.0 months, interquartile range from 21.0 to 47.5 months), 16 MCI patients converted to AD dementia, three MCI patients converted to other types of dementia (1 bvFTD, 1 DLB, and 1 VaD), and the other 25 MCI patients remained stable. In the comparison between persons with MCI converting to AD dementia and persons with MCI remaining stable, there were significant differences in the proportion of PiB positivity (100% vs. 28%, *p* < 0.001), “AD‐typical pattern” (68.75% vs. 28.00%, *p* < 0.05) and hypometabolism in posterior cingulate/precuneus cortex (68.75% vs. 24.00%, *p* < 0.05) on FDG PET (Table S2, Figure 3A,B).

Receiver operating characteristic curve analysis was performed for the positive results on PiB and hypometabolic regions and patterns on FDG with significant differences between MCI persons converting to AD dementia and MCI persons remaining stable (Figure 3C). Compared with regional hypometabolism in posterior cingulate/precuneus cortex (AUC: 0.652, sensitivity: 61.11%, specificity: 69.23%) and “AD‐typical pattern” on FDG PET (AUC: 0.699, sensitivity: 66.67%, specificity: 73.08%), positive results on PiB had a higher AUC of 0.904 (*p* < 0.05), with sensitivity and specificity of 100.00% and 80.77% for discriminating MCI persons converting to AD dementia from those remaining stable.

## 4 DISCUSSION

In this study, we retrospectively analyzed the value of FDG PET combined with PiB PET in the diagnosis of patients with cognitive impairment at memory clinics. Amyloid deposition measured with PiB PET was observed in not only patients clinically diagnosed with AD (72.34%) and MCI (52.27%) but also patients with other types of dementia, such as DLB (66.67%), VaD (66.67%), and FTLD (28.00%). A typical pattern featuring hypometabolism in the temporoparietal cortex and posterior cingulate cortex showed moderate‐to‐high accuracy in predicting amyloid deposition. Both PiB and FDG had effects on diagnostic decisions in patients with dementia. Moreover, although to a lesser degree than PiB, the typical hypometabolic pattern on FDG also effectively predicted MCI conversion to AD dementia.

Similar to previous reports,^24^ 27.66% of patients with clinically diagnosed AD showed an absence of PiB binding in the present study, most of whom had their diagnosis changed to other types of dementia at follow‐up. On the contrary, false‐negative findings are possible and negative PiB results cannot rule out AD diagnosis since PiB PET may not be able to detect more soluble species of Aβ42 or atypical amyloid deposits.^25^ Therefore, a few patients who were PiB negative in our memory clinic still retained an AD diagnosis according to their clinical presentations. However, it has been recognized that the “AD‐like phenocopy” is most prevalent in the oldest patients and may be best explained by a mix of age‐related pathologies (e.g., hippocampal sclerosis, limbic‐predominant age‐related TAR‐DNA binding protein 43 [TDP‐43] encephalopathy, argyrophilic grain disease, or tangle‐predominant dementia) that preferentially target the limbic system, resulting in a memory‐predominant presentation that may be misdiagnosed as AD.^26^, ^27^, ^28^ Currently, for clinically diagnosed AD patients with negative amyloid biomarkers, it is still difficult to determine a definite diagnosis unless neuropathological evidence is obtained.

A fraction of patients with clinically diagnosed FTLD also presented with PiB‐positive results, with 35.71% of bvFTD and 20.00% of PPA (including nfvPPA and svPPA) patients in this study. Postmortem studies showed that ~ 15%–20% of clinically diagnosed bvFTD cases could be caused by AD pathology,^29^, ^30^ also known as “the fvAD.”^31^ Alternatively, comorbid FTLD and AD pathology may be present, with FTLD pathology as the dominant force driving the clinical presentation and amyloid pathology as a by‐product of aging.^32^ It has been suggested that svPPA and nfvPPA are generally caused by FTLD (mainly tau and TDP‐43 proteinopathies),^33^ while lvPPA is mainly caused by AD pathology.^34^, ^35^ However, a considerable proportion of nfvPPA (20%) and svPPA (16%) cases presented Aβ pathology according to a meta‐analysis using individual participant data from 36 centers.^36^ Although CBS caused by AD pathology has been recently recognized and considered an atypical variant of AD,^37^ the CBS patient in this study had negative results on PiB. Accordingly, amyloid PET is useful in identifying AD pathology as the underlying cause or comorbidity in patients with complex presentations, such as PPA variants or CBS,^37^, ^38^ particularly in the condition of DMT targeting Aβ.

We found a large percentage (66.67%) of DLB patients presenting a positive result on PiB PET. This result was supported by previous findings that elevated Aβ on PET scans is a common observation in patients with DLB (up to 60% of patients).^24^, ^39^, ^40^ It has been demonstrated that diffuse plaques primarily comprising Aβ42 are typically abundant in patients with Lewy body disease at autopsy and contribute to elevated PiB binding.^41^ In addition, 66.67% of patients with VaD presented as PiB positive in the present study. These findings supported that concurrent cerebrovascular, Lewy body and AD pathology are very common in elderly patients with dementia.

Compared with amyloid‐negative patients, a higher proportion of amyloid‐positive patients showed significant hypometabolism in the temporoparietal and posterior cingulate/precuneus cortex on FDG PET images, and this effect was even present in patients with MCI. This finding is consistent with previous studies showing that the pattern of temporoparietal and posterior cingulate/precuneus cortex hypometabolism is a reliable marker of AD.^42^, ^43^ We found that the “AD‐typical pattern” on FDG PET images is a useful marker in the diagnosis of both AD and MCI due to AD, with a moderate‐to‐high accuracy in identifying amyloid deposition measured with PiB in this study. Previous studies reported that FDG PET results could significantly improve the diagnostic accuracy from 77% to 90% for AD and from 85% to 94% for FTLD among dementia patients with uncertain diagnosis.^44^

In this study, the initial diagnosis was changed after considering PET results in 32.17% of patients with dementia. When combined with PiB PET results, FDG PET (e.g., hypometabolism in different brain regions or hypometabolic patterns) showed an additional influence on diagnosis decisions at follow‐up. This finding is supported by current diagnostic criteria for several types of neurodegenerative dementia,^4^, ^5^, ^6^, ^7^ which recommend FDG PET as a valuable neuroimaging biomarker.

In this study, amyloid positivity was 52.27% in persons with MCI, which is consistent with previous findings in other studies of memory clinic patients.^45^, ^46^, ^47^ Amyloid positivity was 26.5% in the nonamnestic MCI (naMCI) group and 64.7% in the amnestic MCI (aMCI) group in a previous study.^47^ We did not differentiate these two subtypes in our participants, although most patients were classified as aMCI based on medical records. Moreover, consistent a meta‐analysis showing that amyloid PET had a sensitivity of 93% and a specificity of 56% for predicting MCI conversion to AD,^48^ PiB results showed a high value in predicting AD dementia conversion from MCI at long‐term follow‐up with a median time of 32 months. Moreover, the “AD‐typical pattern” of hypometabolism on FDG PET also predicted AD conversion in persons with MCI. However, the sensitivity and specificity of FDG PET varied from 25% to 100% and from 15% to 100% for predicting conversion from MCI to AD in previous studies.^49^ Another study found that FDG showed higher specificity (100% vs. 62%) but lower sensitivity (79% vs. 100%) than PiB PET in predicting AD conversion.^50^ The observed variability was likely due to variability in demographic variables, follow‐up durations, and more importantly, the heterogeneity in the analysis methodology to define PiB positivity and FDG regions and patterns. In addition, as a downstream marker for neuronal injury, changes in FDG likely appear later than amyloid deposition on PET and therefore show a relatively high specificity and low sensitivity in predicting AD conversion.

There were several limitations in the present study. First, participants in this study, who received PET scans sometimes for specific reasons, such as diagnostic uncertainty, could not represent the entire population of patients, although PET scans are a current day‐to‐day practice in memory clinics. Second, since the diagnosis of all participants was based on the clinical diagnostic criteria only and lacked pathological confirmation, it is still impossible to validate the diagnostic accuracy of FDG PET in patients with dementia. Third, since this was a retrospective study, we did not follow up all dementia patients in the long term. These patients' diagnosis could have changed further during long‐term follow‐up. Finally, although the PET images were analyzed and read by two experienced radiologists for a consensus, the interpretations depended heavily on individual experience and training; additionally, the radiologists were not completely blinded to clinical information.

## 5 CONCLUSION

Amyloid deposition measured with PiB PET could be observed in clinically diagnosed patients with many types of dementia, such as DLB, VaD, and FTLD, in addition to AD and MCI. A typical pattern featuring temporoparietal and posterior cingulate/precuneus cortex hypometabolism visually identified on FDG PET could predict amyloid deposition and was also helpful in the diagnosis of patients with dementia and predicting AD dementia conversion for persons with MCI in addition to PiB PET.

## AUTHOR CONTRIBUTIONS

Fang Liu and Yudi Shi collected the data. Fang Liu, Yudi Shi, and Huifeng Chen analyzed the data. Qiuyan Wu prepared Figures 1, 2, 3. Ying Wang and Li Cai performed the PET procedure and evaluated the images. Fang Liu wrote the manuscript. Nan Zhang conceived and designed the study and reviewed and revised the manuscript.

## FUNDING INFORMATION

Tianjin Health Science and Technology Project (ZC20230) and Tianjin Key Medical Discipline (Specialty) Construction Project (TJYXZDXK‐004A).

## CONFLICT OF INTEREST STATEMENT

All authors declare that they have no competing interests.

## PATIENT CONSENT STATEMENT

This study was approved by the Ethics Committees of Tianjin Medical University General Hospital. Informed consent was obtained from all individual participants included in the study.

## Supporting information


**Table S1.** Differences in hypometabolic regions on FDG PET between amyloid‐positive and amyloid‐negative patients with dementia or MCI
**Table S2.** Differences in hypometabolic regions on FDG PET between MCI patients converting to AD dementia and MCI patients remaining stable

## Data Availability

The raw data supporting the conclusion of this manuscript will be made available by the corresponding author, without undue reservation, to any qualified researcher.

## References

[1] Estimation of the global prevalence of dementia in 2019 and forecasted prevalence in 2050: an analysis for the global burden of disease study 2019. Lancet Public Health. 2022;7(2):e105‐e125. 34998485 10.1016/S2468-2667(21)00249-8 PMC8810394

[2] Kawakatsu S , Kobayashi R , Hayashi H . Typical and atypical appearance of early‐onset Alzheimer's disease: a clinical, neuroimaging and neuropathological study. Neuropathology: Official Journal of the Japanese Society of Neuropathology. 2017;37(2):150‐173. 28093855 10.1111/neup.12364

[3] Galton CJ , Patterson K , Xuereb JH , Hodges JR . Atypical and typical presentations of Alzheimer's disease: a clinical, neuropsychological, neuroimaging and pathological study of 13 cases. Brain. 2000;123(Pt 3):484‐498. 10686172 10.1093/brain/123.3.484

[4] Jack CR Jr , Bennett DA , Blennow K , et al. NIA‐AA research framework: toward a biological definition of Alzheimer's disease. Alzheimers Dement. 2018;14(4):535‐562. 29653606 10.1016/j.jalz.2018.02.018 PMC5958625

[5] Rascovsky K , Hodges JR , Knopman D , et al. Sensitivity of revised diagnostic criteria for the behavioural variant of frontotemporal dementia. Brain. 2011;134(Pt 9):2456‐2477. 21810890 10.1093/brain/awr179 PMC3170532

[6] Gorno‐Tempini ML , Hillis AE , Weintraub S , et al. Classification of primary progressive aphasia and its variants. Neurology. 2011;76(11):1006‐1014. 21325651 10.1212/WNL.0b013e31821103e6 PMC3059138

[7] McKeith IG , Boeve BF , Dickson DW , et al. Diagnosis and management of dementia with Lewy bodies: fourth consensus report of the DLB consortium. Neurology. 2017;89(1):88‐100. 28592453 10.1212/WNL.0000000000004058 PMC5496518

[8] Klunk WE , Engler H , Nordberg A , et al. Imaging brain amyloid in Alzheimer's disease with Pittsburgh compound‐B. Ann Neurol. 2004;55(3):306‐319. 14991808 10.1002/ana.20009

[9] Ikonomovic MD , Buckley CJ , Abrahamson EE , et al. Post‐mortem analyses of PiB and flutemetamol in diffuse and cored amyloid‐β plaques in Alzheimer's disease. Acta Neuropathol. 2020;140(4):463‐476. 32772265 10.1007/s00401-020-02175-1 PMC7498488

[10] Ikonomovic MD , Klunk WE , Abrahamson EE , et al. Post‐mortem correlates of in vivo PiB‐PET amyloid imaging in a typical case of Alzheimer's disease. Brain. 2008;131(Pt 6):1630‐1645. 18339640 10.1093/brain/awn016 PMC2408940

[11] Zimmer ER , Parent MJ , Souza DG , et al. [(18)F]FDG PET signal is driven by astroglial glutamate transport. Nat Neurosci. 2017;20(3):393‐395. 28135241 10.1038/nn.4492 PMC5378483

[12] Attwell D , Laughlin SB . An energy budget for signaling in the grey matter of the brain. J Cereb Blood Flow Metab. 2001;21(10):1133‐1145. 11598490 10.1097/00004647-200110000-00001

[13] Nestor PJ , Altomare D , Festari C , et al. Clinical utility of FDG‐PET for the differential diagnosis among the main forms of dementia. Eur J Nucl Med Mol Imaging. 2018;45(9):1509‐1525. 29736698 10.1007/s00259-018-4035-y

[14] Lesman‐Segev OH , La Joie R , Iaccarino L , et al. Diagnostic accuracy of amyloid versus (18) F‐Fluorodeoxyglucose positron emission tomography in autopsy‐confirmed dementia. Ann Neurol. 2021;89(2):389‐401. 33219525 10.1002/ana.25968 PMC7856004

[15] Ossenkoppele R , Prins ND , Pijnenburg YA , et al. Impact of molecular imaging on the diagnostic process in a memory clinic. Alzheimers Dement. 2013;9(4):414‐421. 23164552 10.1016/j.jalz.2012.07.003

[16] McKhann GM , Knopman DS , Chertkow H , et al. The diagnosis of dementia due to Alzheimer's disease: recommendations from the National Institute on Aging‐Alzheimer's Association workgroups on diagnostic guidelines for Alzheimer's disease. Alzheimers Dement. 2011;7(3):263‐269. 21514250 10.1016/j.jalz.2011.03.005 PMC3312024

[17] Petersen RC . Mild cognitive impairment as a diagnostic entity. J Intern Med. 2004;256(3):183‐194. 15324362 10.1111/j.1365-2796.2004.01388.x

[18] McKeith IG , Dickson DW , Lowe J , et al. Diagnosis and management of dementia with Lewy bodies: third report of the DLB consortium. Neurology. 2005;65(12):1863‐1872. 16237129 10.1212/01.wnl.0000187889.17253.b1

[19] Armstrong MJ , Litvan I , Lang AE , et al. Criteria for the diagnosis of corticobasal degeneration. Neurology. 2013;80(5):496‐503. 23359374 10.1212/WNL.0b013e31827f0fd1 PMC3590050

[20] Sachdev P , Kalaria R , O'Brien J , et al. Diagnostic criteria for vascular cognitive disorders: a VASCOG statement. Alzheimer Dis Assoc Disord. 2014;28(3):206‐218. 24632990 10.1097/WAD.0000000000000034 PMC4139434

[21] American Psychiatric Association , ed. Diagnostic and Statistical Manual of Mental Disorders. 5th ed. American Psychiatric Association; 2013.

[22] Wang Y , Shi Z , Zhang N , et al. Spatial patterns of Hypometabolism and amyloid deposition in variants of Alzheimer's disease corresponding to brain networks: a prospective cohort study. Mol Imaging Biol. 2019;21(1):140‐148. 29869063 10.1007/s11307-018-1219-6

[23] Zhang N , Zhang L , Li Y , et al. Urine AD7c‐NTP predicts amyloid deposition and symptom of agitation in patients with Alzheimer's disease and mild cognitive impairment. J Alzheimers Dis. 2017;60(1):87‐95. 28777752 10.3233/JAD-170383 PMC5611795

[24] Ossenkoppele R , Jansen WJ , Rabinovici GD , et al. Prevalence of amyloid PET positivity in dementia syndromes: a meta‐analysis. Jama. 2015;313(19):1939‐1949. 25988463 10.1001/jama.2015.4669 PMC4517678

[25] Leinonen V , Alafuzoff I , Aalto S , et al. Assessment of beta‐amyloid in a frontal cortical brain biopsy specimen and by positron emission tomography with carbon 11‐labeled Pittsburgh compound B. Arch Neurol. 2008;65(10):1304‐1309. 18695050 10.1001/archneur.65.10.noc80013

[26] Barkhof F , Polvikoski TM , van Straaten EC , et al. The significance of medial temporal lobe atrophy: a postmortem MRI study in the very old. Neurology. 2007;69(15):1521‐1527. 17923614 10.1212/01.wnl.0000277459.83543.99

[27] Serrano‐Pozo A , Qian J , Monsell SE , et al. Mild to moderate Alzheimer dementia with insufficient neuropathological changes. Ann Neurol. 2014;75(4):597‐601. 24585367 10.1002/ana.24125 PMC4016558

[28] Crary JF , Trojanowski JQ , Schneider JA , et al. Primary age‐related tauopathy (PART): a common pathology associated with human aging. Acta Neuropathol. 2014;128(6):755‐766. 25348064 10.1007/s00401-014-1349-0 PMC4257842

[29] Perry DC , Brown JA , Possin KL , et al. Clinicopathological correlations in behavioural variant frontotemporal dementia. Brain. 2017;140(12):3329‐3345. 29053860 10.1093/brain/awx254 PMC5841140

[30] Forman MS , Farmer J , Johnson JK , et al. Frontotemporal dementia: clinicopathological correlations. Ann Neurol. 2006;59(6):952‐962. 16718704 10.1002/ana.20873 PMC2629792

[31] Ossenkoppele R , Pijnenburg YA , Perry DC , et al. The behavioural/dysexecutive variant of Alzheimer's disease: clinical, neuroimaging and pathological features. Brain. 2015;138(Pt 9):2732‐2749. 26141491 10.1093/brain/awv191 PMC4623840

[32] Pennington C , Marini L , Coulthard E , Love S . Mixed neuropathology in frontotemporal lobar degeneration. Amyotrophic Lateral Sclerosis & Frontotemporal Degeneration. 2020;21(3–4):301‐308. 32116039 10.1080/21678421.2020.1733019

[33] Mackenzie IR , Neumann M , Bigio EH , et al. Nomenclature and nosology for neuropathologic subtypes of frontotemporal lobar degeneration: an update. Acta Neuropathol. 2010;119(1):1‐4. 19924424 10.1007/s00401-009-0612-2 PMC2799633

[34] Mesulam MM , Weintraub S , Rogalski EJ , Wieneke C , Geula C , Bigio EH . Asymmetry and heterogeneity of Alzheimer's and frontotemporal pathology in primary progressive aphasia. Brain. 2014;137(Pt 4):1176‐1192. 24574501 10.1093/brain/awu024 PMC3959558

[35] Harris JM , Gall C , Thompson JC , et al. Classification and pathology of primary progressive aphasia. Neurology. 2013;81(21):1832‐1839. 24142474 10.1212/01.wnl.0000436070.28137.7b

[36] Bergeron D , Gorno‐Tempini ML , Rabinovici GD , et al. Prevalence of amyloid‐β pathology in distinct variants of primary progressive aphasia. Ann Neurol. 2018;84(5):729‐740. 30255971 10.1002/ana.25333 PMC6354051

[37] Dubois B , Villain N , Frisoni GB , et al. Clinical diagnosis of Alzheimer's disease: recommendations of the international working group. The Lancet Neurology. 2021;20(6):484‐496. 33933186 10.1016/S1474-4422(21)00066-1 PMC8339877

[38] Bouwman F , Orini S , Gandolfo F , et al. Diagnostic utility of FDG‐PET in the differential diagnosis between different forms of primary progressive aphasia. Eur J Nucl Med Mol Imaging. 2018;45(9):1526‐1533. 29744573 10.1007/s00259-018-4034-z PMC6061469

[39] Gomperts SN , Rentz DM , Moran E , et al. Imaging amyloid deposition in Lewy body diseases. Neurology. 2008;71(12):903‐910. 18794492 10.1212/01.wnl.0000326146.60732.d6 PMC2637553

[40] Gao ZB , Wang W , Zhao XL , et al. Multi‐modality molecular imaging characteristics of dementia with Lewy bodies. J Int Med Res. 2018;46(6):2317‐2326. 29619853 10.1177/0300060518764749 PMC6023065

[41] Kantarci K , Lowe VJ , Chen Q , et al. β‐Amyloid PET and neuropathology in dementia with Lewy bodies. Neurology. 2020;94(3):e282‐e291. 31862783 10.1212/WNL.0000000000008818 PMC7108811

[42] Foster NL , Heidebrink JL , Clark CM , et al. FDG‐PET improves accuracy in distinguishing frontotemporal dementia and Alzheimer's disease. Brain. 2007;130(Pt 10):2616‐2635. 17704526 10.1093/brain/awm177

[43] Mosconi L . Brain glucose metabolism in the early and specific diagnosis of Alzheimer's disease. FDG‐PET studies in MCI and AD. Eur J Nucl Med Mol Imaging. 2005;32(4):486‐510. 15747152 10.1007/s00259-005-1762-7

[44] Perini G , Rodriguez‐Vieitez E , Kadir A , Sala A , Savitcheva I , Nordberg A . Clinical impact of (18)F‐FDG‐PET among memory clinic patients with uncertain diagnosis. Eur J Nucl Med Mol Imaging. 2021;48(2):612‐622. 32734458 10.1007/s00259-020-04969-7 PMC7835147

[45] Rabinovici GD , Gatsonis C , Apgar C , et al. Association of Amyloid Positron Emission Tomography with Subsequent Change in clinical management among Medicare beneficiaries with mild cognitive impairment or dementia. Jama. 2019;321(13):1286‐1294. 30938796 10.1001/jama.2019.2000 PMC6450276

[46] Tomadesso C , de La Sayette V , de Flores R , et al. Neuropsychology and neuroimaging profiles of amyloid‐positive versus amyloid‐negative amnestic mild cognitive impairment patients. Alzheimer's & Dementia (Amsterdam, Netherlands). 2018;10:269‐277. 10.1016/j.dadm.2018.02.008 PMC5956939 29780872

[47] Lee SH , Lee JH , Byun MS , et al. Comparison of amyloid positivity rate and accumulation pattern between amnestic and non‐amnestic type mild cognitive impairment. Psychiatry Investig. 2020;17(6):603‐607. 10.30773/pi.2020.0063 PMC7324742 32517418

[48] Zhang S , Han D , Tan X , Feng J , Guo Y , Ding Y . Diagnostic accuracy of 18 F‐FDG and 11 C‐PIB‐PET for prediction of short‐term conversion to Alzheimer's disease in subjects with mild cognitive impairment. Int J Clin Pract. 2012;66(2):185‐198. 22257044 10.1111/j.1742-1241.2011.02845.x

[49] Smailagic N , Vacante M , Hyde C , Martin S , Ukoumunne O , Sachpekidis C . ^18^F‐FDG PET for the early diagnosis of Alzheimer's disease dementia and other dementias in people with mild cognitive impairment (MCI). Cochrane Database Syst Rev. 2015;1(1):Cd010632. 25629415 10.1002/14651858.CD010632.pub2 PMC7081123

[50] Iaccarino L , Chiotis K , Alongi P , et al. A cross‐validation of FDG‐ and amyloid‐PET biomarkers in mild cognitive impairment for the risk prediction to dementia due to Alzheimer's disease in a clinical setting. J Alzheimers Dis. 2017;59(2):603‐614. 28671117 10.3233/JAD-170158

