# Supplementary material for: The value of FDG combined with PiB PET in the diagnosis of patients with cognitive impairment in a memory clinic
Source: CNS Neurosci Ther. 2023 Aug 21;30(2):e14418. doi: 10.1111/cns.14418 (PMC10848040; doi:10.1111/cns.14418)
Supplement: Supplementary file 1 — Table S1. Differences in hypometabolic regions on FDG PET between amyloid‐positive and amyloid‐negative patients with dementia or MCI Table S2. Differences in hypometabolic regions on FDG PET between MCI patients converting to AD dementia and MCI patients remaining stable [file CNS-30-e14418-s001.docx]

**Supplementary Materials**

**Table S1.** Differences in hypometabolic regions on FDG PET between amyloid-positive and amyloid-negative patients with dementia or MCI

|  | Patients with dementia | | | | Patients with MCI | | | |
| --- | --- | --- | --- | --- | --- | --- | --- | --- |
|  | PiB+ | PiB- | *χ2* | *P* | PiB+ | PiB- | *χ2* | *P* |
|  | (n=84) | (n=59) |  |  | (n=23) | (n=21) |  |  |
| Medial frontal lobes | 59 (70.24%) | 45 (76.27%) | 0.635 | 0.425 | 15 (65.22%) | 15 (71.43%) | 0.195 | 0.659 |
| Lateral frontal lobes | 74 (88.10%) | 53 (89.83%) | 0.105 | 0.746 | 18 (78.26%) | 19 (90.48%) | 0.482 | 0.488^a^ |
| Anterior temporal cortex | 25 (29.76%) | 20 (33.90%) | 0.275 | 0.600 | 4 (17.39%) | 4 (19.05%) | 0.000 | 1.000^a^ |
| Temporoparietal cortex | 42 (50.00%) | 5 (8.47%) | 27.085 | <0.001 | 5 (21.74%) | 0 | 3.218 | 0.073^a^ |
| Posterior cingulate/precuneus cortex | 69 (82.14%) | 18 (30.51%) | 38.783 | <0.001 | 13 (56.52%) | 6 (28.57%) | 3.495 | 0.062 |
| Occipital lobes | 14 (16.67%) | 6 (10.17%) | 1.216 | 0.270 | 1 (4.35%) | 1 (4.67%) | / | 1.000^b^ |

Data are presented as No. (%)

^a^ were determined with Yates’s corrections for continuity

^b^ was determined with Fisher’s exact test

Abbreviation: FDG, fluorodeoxyglucose; MCI, mild cognitive impairment; PET, positron emission tomography; PiB, Pittsburgh compound B

**Table S2.** Differences in hypometabolic regions on FDG PET between MCI patients converting to AD dementia and MCI patients remaining stable

|  | MCI conversion (n=16) | MCI stable  (n=25) | *χ2* | *P* |
| --- | --- | --- | --- | --- |
| Medial frontal lobes | 11 (68.75%) | 16 (64.00%) | 0.098 | 0.754 |
| Lateral frontal lobes | 12 (75.00%) | 22 (88.00%) | 0.427 | 0.513^a^ |
| Anterior temporal cortex | 4 (25.00%) | 3 (12.00%) | 0.427 | 0.513^a^ |
| Temporoparietal cortex | 4 (25.00%) | 1 (4.00%) | 2.296 | 0.130^a^ |
| Posterior cingulate/precuneus cortex | 11 (68.75%) | 6 (24.00%) | 8.050 | 0.005 |
| Occipital lobe | 1 (6.25%) | 0 | / | 0.390^b^ |

Data are presented as No. (%)

^a^ were determined with Yates’s corrections for continuity

^b^ was determined with Fisher’s exact test

Abbreviation: AD, Alzheimer’s disease; FDG, fluorodeoxyglucose; MCI, mild cognitive impairment; PET, positron emission tomography
